# A participatory and comprehensive intervention to improve violence prevention in two high-risk occupations: effect and process evaluation of a stepped wedge cluster randomised trial

**DOI:** 10.1186/s12889-024-18527-5

**Published:** 2024-04-15

**Authors:** Lars Peter Andersen, S. Jaspers, D. Andersen, I. Karlsen, B. Aust

**Affiliations:** 1 Department of Occupational Medicine, Danish Ramazzini Centre, University Research Clinic, Goedstrup Hospital, Hospitalsparken 15, 7400 Herning, Denmark; 2 The National Research Centre for the Working Environment, Lersø Parkallé 105, 2100 Copenhagen Ø, Denmark

**Keywords:** Work-related violence and threats, Employee participation, Comprehensive violence prevention, Implementation

## Abstract

**Background:**

Work-related violence committed by clients, patients, and customers represents a major occupational health risk for employees that needs to be reduced.

**Methods:**

We tested a comprehensive violence prevention intervention involving active participation of both employees and managers in the Prison and Probation Service (PPS) and on psychiatric wards in Denmark. We used a stepped wedge cluster randomised controlled trial design. We measured the degree of implementation of the intervention by registration of fidelity, reach, and dose and used a mixed-effects regression analysis to estimate the effects of the intervention.

**Results:**

We recruited 16 work units for the intervention, but three work units dropped out. The average implementation rate was 73%. In the psychiatric wards, the intervention led to statistically significant improvements in the primary outcome (an increase in the degree to which managers and employees continuously work on violence prevention practices based on their registration and experiences), but none statistically significant improvements in any of the secondary outcomes. In the PPS units, the intervention did not lead to a statistically significant improvement in the primary outcome, but to statistically significant improvements in three secondary outcomes.

**Conclusion:**

Most work units were able to carry out the intervention as planned. The intervention showed mixed results regarding the primary outcome. Nevertheless, the results indicate improvements also in the sector where a change in the primary outcome was not achieved. The results point at that a participatory and comprehensive approach could be a viable way of working with violence prevention in high-risk workplaces.

**Trial registration:**

ISRCTN86993466: 20/12/2017

## Introduction

Work-related violence and threats continue to be a major occupational risk for many employees [1]. The International Labour Office defines work-related violence as “any action, incident or behaviour that departs from reasonable conduct in which a person is assaulted, threatened, harmed or injured in the course of, or as a direct result of, his or her work” [2]. Jobs that involve client contact, working alone or in isolated areas, and providing services or care increase the risk of being exposed to work-related violence [3, 4]. Workplace violence where the perpetrator is e.g., a client that has a legitimate relationship with the workplace is called type II violence or client-on-worker violence [5]. Certain sectors have elevated risks for this type of violence, among them psychiatric wards and prisons [6–11].

Work-related violence has consequences for employees’ physical and psychological health and can lead to reduced organisational commitment, absenteeism and turnover [12]. Working on psychiatric wards and in prisons places employees at a high risk of developing depressive symptoms and PTSD following exposure to work-related violence and threats [13–16].

### Risk factors for work-related violence

Client-on-worker violence is a complex phenomenon influenced by individual, situational, structural, organizational, and cultural factors [17] and it is often the endpoint of a process with multiple factors and failures [18]. Work-related violence and threats occur in a specific situation, but broader situational and structural factors shape the context and influence the circumstances prior to and following these incidents [19]. To address the complexity of violence prevention, researchers are increasingly calling for comprehensive interventions [17, 20, 21].

### Comprehensive interventions for violence prevention

So far, only a few interventions have tested a comprehensive approach for violence prevention [22–25]. Arnetz and colleagues [24] examined a participatory action approach focused on continuous quality improvement based on registration of violent episodes. Results showed that six months post-intervention, incident rate ratios of violent events were significantly lower in intervention units than in control units [24]. The participatory approach in this study may have ensured that the intervention was adapted to local work procedures, increasing the chances of finding sustainable solutions.

Another comprehensive intervention is Safewards, an internationally adopted approach to reduce conflict and containment in healthcare settings, consisting of ten possible interventions [26]. The success of Safewards depends on adequate preparation of staff, support during implementation, and adapting training materials to the local context [27, 28]. However, resistance to change may impede implementation of the intervention [22, 27, 28], limiting its potential effects [29].

The above mentioned studies serve as strong indicators for the potential benefits of comprehensive and participatory violence preventive efforts. The present study builds on these experiences. It is based on a comprehensive and participatory approach, while also addressing the challenges found in the existing studies, namely tailoring the intervention to the local context and assessing the implementation as a prerequisite for potential effects.

### Theoretical framework and programme theory of the study

This study is based on a theory from an adjacent field of research: the theory of integrated accident prevention by David D. DeJoy [30]. Central to this theory is a participatory problem-solving process involving both leaders and workers. The theory integrates interventions at the management level focusing on culture change with interventions at the employee level focusing on behaviour change, thereby facilitating the two levels to jointly identify relevant safety problems and possible solutions. By integrating the two approaches, it is assumed that a comprehensive approach to prevention can be achieved. An important aspect is the problem-solving process of using data to design and evaluate preventive actions. Intervention studies based on this approach have documented improved safety and employees’ perceptions of safety [31, 32].

Even though the theoretical framework was originally developed to prevent accidents at work and increase occupational safety, prevention of work-related violence can be considered as part of occupational safety. Assuming that the application of DeJoy’s integrated approach could also increase safety in relation to work-related violence, we developed the Integrated Violence Prevention (IVP) intervention. The behaviour based problem-solving approach is in this case focused on the continuous use of registrations of violent episodes. The use of registrations enables the work units to identify patterns of violence and threats and based on that develop adequate preventive measures. It is expected that the intervention will lead to continuous adjustment of the work units’ violence prevention.

For further details on the intervention design see Jaspers et al., 2019 [33].

### Aim of the study

The aim was to evaluate the effects of the IVP intervention on violence prevention practice and to assess the degree to which the intervention was implemented.

More specifically, we aimed to test the following hypotheses:

1. The intervention will lead to a significant increase in the degree to which managers and employees continuously work on violence prevention practices based on their registration and experiences (main outcome).
2. Work units with a higher degree of implementation will have a greater increase in the main outcome than work units with a lower degree of implementation.
3. At work units with a high degree of implementation, there will be a greater increase in the secondary outcomes: (a) cooperation between line managers and employees; (b) attention to violence prevention; (c) the number of actions taken to prevent violence and threats; (d) violence prevention practices; (e) the violence prevention climate; (f) the employees’ self and collective efficacy in violence prevention; and (g) the employees’ sense of safety at work.
4. Work units with a higher baseline score of main and secondary outcomes will experience smaller improvements post intervention due to a ceiling effect, even when the implementation degree is high.

## Methods

### Recruitment of work units and participants

Between April and June 2017, 16 work units were recruited, eight from psychiatric hospitals and eight from the Prison and Probation Service (PPS). We recruited the psychiatric wards by contacting either the top management or the work environment coordinator.

In the PPS the top management encouraged all prisons and detention centres in Denmark (in total 52) to participate. Two of the eight work units in the PPS responded late to this call and were only recruited in October 2017, one month after the first baseline measurement in psychiatry. This was taken into account in the evaluation.

Work units were eligible if they (1) had more than ten employees; (2) did not share a line manager; (3) were not ambulatory departments (for psychiatric units); and (4) currently were not involved in an intervention that shared some characteristics with our intervention.

Altogether 430 employees participated at baseline, 249 employees from psychiatric wards and 181 employees from the PPS.

### The Integrated Violence Prevention Intervention

**Fig. 1:**
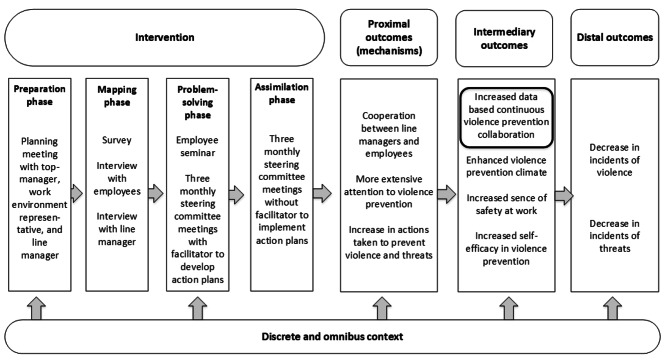
Illustration of the study’s intervention, mechanism and outcomes

The IVP intervention consisted of four phases: (1) Preparation, (2) Mapping of existing violence prevention practices, (3) Problem-solving process, and (4) Assimilation (see Fig. 1).

**Fig. 2:**
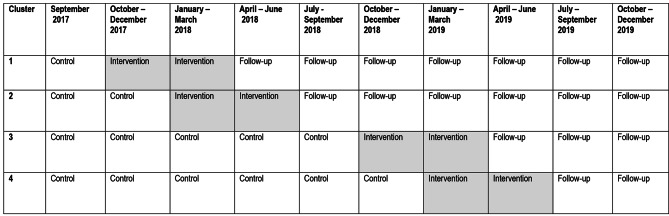
Illustration of the study’s cluster-randomized controlled trial with a stepped wedge design

The intervention was carried out from September 2017 to June 2019. Following a stepped wedge design, we grouped the participating work units into clusters that entered the intervention at different times (see Fig. 2). The intervention included a staff seminar for all unit members, steering group meetings involving management and selected personnel, and individual coaching sessions for the manager. After the first cluster of work units had received the intervention, some adjustments in the intervention activities were made to better fit the resources of the work units while retaining the core elements of the intervention to ensure comparability.

Below, we describe the adjusted version of the intervention that was used by the majority of the participating work units.

#### Preparation

Before starting the intervention, we met with the line manager, the immediate manager, and the occupational health and safety representative (OHS) at each work unit to plan the implementation of the intervention and ensure the necessary resources.

#### Mapping of violence prevention practices

We assessed the existing violence prevention practices before the intervention using a questionnaire, conducting an interview with the line manager and a focus group interview with employees.

#### Problem-solving process

The problem-solving process was initiated with a steering group meeting at each work unit where results from the mapping phase were presented. The steering group consisted of the line manager, the OHS representative, and two to three employees. To involve all employees in the process, a three-hour seminar was held one month later with (ideally) all employees and the line manager. The results from the mapping phase were presented, and the employees and their line manager identified the most relevant challenges for violence prevention at their work unit.

This was followed by discussions where participants were prompted to identify actions on the individual, group, and organisational levels to facilitate a systematic and comprehensive approach to prevention. The suggested actions were collected and communicated to the steering group.

After the seminar, the steering group met once a month to work with the suggested actions. The meetings were facilitated by members of the research team. The steering group discussed and prioritized the suggested actions, and developed action plans with an appointed person in charge of execution and specific follow-up dates.

#### Assimilation phase

To encourage the work units to assimilate the intervention concept, the work units were asked to continue to implement action plans and hold self-facilitated monthly steering group meetings three month post intervention.

### Evaluation design

The effect evaluation was carried out as a cluster-randomised controlled trial with a stepped wedge design. This design involves random and sequential crossover of clusters from control to intervention until all clusters have received the intervention, meaning that all participating work units receive the intervention, but at different time points (steps). Before the intervention, the work units function as control work units, after the intervention, the work units contribute with follow-up measurements. Groups of work units (clusters) were randomised to different starting points.

### Randomisation

The two sectors were randomised separately. Work units were randomised to four different starting dates (October 2017, January 2018, October 2018, and January 2019). At each starting date, the intervention was implemented in two psychiatric work units and two work units in the PPS. Due to the delayed recruitment of PPS work units, they were randomised to the intervention on only the last three starting dates. To diminish the spill over effect across work units that were located in the same buildings, the work units were stratified by geography, meaning that work units that were geographically close to each other received the intervention at the same time or shortly after.

An independent statistician developed and performed a computer-generated randomisation using SAS for Windows statistical software (SAS Institute, Cary, NC, USA). The sequence was concealed for the researchers in a computer folder with code-restricted access. Blinding of participants was not possible owing to the nature of the participatory approach.

### Primary and secondary outcomes

The primary and secondary outcomes were chosen in line with the programme theory developed for the study. The primary outcome was defined in line with hypothesis 1 as an increase in the degree to which managers and employees continuously work on violence prevention practices based on their registration and experiences.

The main outcome is marked in Fig. 1 by a thick line.

Systematic registration of violent and threatening incidents is crucial for the assessment of risks and the identification of patterns associated with frequent episodes. Our assumption was that using the information from the registered incidents of violent or threatening episodes, the work units could develop action plans that help reduce the risk situation they identified.

We deliberately chose not to include self-reported frequencies of violence and threats as primary outcomes, but as secondary and distal outcomes (see Fig. 1). This decision was motivated by several concerns, among them that violence prevention interventions can cause an increase in the reporting of violence and threats due to raised awareness [34–36]. Thus, using the frequency of work-related violence and threats as outcome measure may be unreliable due to reporting bias. Furthermore, although we expected that the intervention would over time have an effect on the incidence of violence and threats, we did not expect that this change would happen during the follow-up period.

### Questionnaire for the effect evaluation

No validated instrument was available that could be used to assess the outcomes we aimed to change with the intervention. An exception was the Violence Prevention Climate Scale, a validated instrument that has demonstrated association with exposure to violence and threats [37]. We chose six items, two from each sub-scale and tailored the wording. Additionally, we included a self-constructed item asking about continuous effort to prevent violence, which was analysed separately. All other outcome measures were self-constructed. To ensure face validity, the wording of the items was discussed with experts working in psychiatry and in the PPS. Table 1 gives an overview of all outcomes measures and the Chronbach’s alpha for the violence prevention climate scale. All other items were used as single items. For details, see Table 1.

**Table 1:** Overview of construct and topics related to violence prevention used in the study

Construct and topics related to violence prevention	Items	Answers	Chronbach’s Alpha
**Primary outcome**			
Data based continuous violence prevention collaboration	To what extent are our violence prevention efforts continuously adjusted as a result of registrations and shared experiences?	Answers range from 0 to 10. “0” signifies “Not at all”; “10” signifies “To a great extent”.	NA
**Secondary outcomes**			
Cooperation between line managers and employees	To what extent do the line manager and employees cooperate on the prevention of violence and threats?	Answers range from 0 to 10. “0” signifies “Not at all”; “10” signifies “To a great extent”.	NA
Attention to violence prevention	To what extent does your line manager prioritise violence prevention? and To what extent does the work environment organisation prioritise violence prevention?	Answers range from 0 to 10. “0” signifies “Not at all”; “10” signifies “To a great extent”.	NA (used as single items)
Actions taken to prevent violence and threats	Have there been improvements related to the prevention of violence and threats during the last 3 months?	Answers range from 0 to 10. “0” signifies “Not at all”; “10” signifies “To a great extent”.	NA
Violence prevention practices in your work unit	To what extent are guidelines on violence prevention carried out in practice at your workplace by your line manager? and To what extent are guidelines on violence prevention carried out in practice at your workplace by the employees?	Answers range from 0 to 10. “0” signifies “Not at all”; “10” signifies “To a great extent”.	NA (used as single items)
Violence prevention climate scale	(a) My line manager quickly responds to episodes of violence. (b) Reports of violence from other employees are taken seriously by my line manager. (c) In my unit, you are trained in violence prevention policies and procedures. (d) In my unit, employees are informed about potential violence hazards. (e) In my unit, whenever pressure builds up, the preference is to do the job as fast as possible, even if it means compromising violence prevention. (f) Human resource shortage and/or staff composition undermines violence prevention standards.	Answer categories: “Disagree very much”, “Disagree moderately”, “Disagree slightly”, “Agree slightly”, “Agree moderately”, “Agree very much”.	Cronbach alpha for this scale was 0.68 (psychiatry) and 0.69 (PPS).
Additional item about violence prevention climate	To the above items, we added the following: g) We are a unit that continuously makes an effort to prevent violence and threats. (However, this item was analysed separately and not as part of the scale.)	Answer categories: “Disagree very much”, “Disagree moderately”, “Disagree slightly”, “Agree slightly”, “Agree moderately”, “Agree very much”.	NA (used as single item)
Self-efficacy in violence prevention	To what extent do you have confidence in your colleagues’ competences to prevent violence and threats? and To what extent do you feel capable to deal with and prevent violence and threats?	Answers range from 0 to 10. “0” signifies “Not at all”; “10” signifies “To a great extent”.	NA (used as single items)
Sense of safety at work	Do you feel safe when you are at work?	Answers range from 0 to 10. “0” signifies “Not at all”; “10” signifies “To a great extent”.	NA
Prevalence of violence and threats	Within the last 3 months have you been exposed to physical violence in your department? and Within the last 3 months have you been exposed to threats in your department?	“Yes, daily”; “Yes, weekly”; “Yes, monthly”; “Yes, from time to time”; “No, never”	NA (used as single items)

The questionnaire was distributed electronically every third month during the entire intervention period. The first cluster received questionnaires every third month from one month prior to the intervention until 21 months after the intervention had ended. The last cluster received questionnaires every third month from 16 months prior to the intervention until six months after the intervention had ended (see Fig. 2). Thus, the intervention was measured at ten time points.

The questionnaire was filled out by those employed in the work unit at each of the ten time points. Therefore, all participants were not the same throughout the study.

### Process evaluation measures

The degree of implementation was measured by documenting fidelity, reach, and dose received for each intervention activity. The measurement scheme we used was inspired by a scheme developed for another complex organisational intervention study [38]. We operationalised the reach of the intervention by documenting who (line manager or employee) and how many participants attended the intervention activities. Fidelity was operationalised by documenting to which degree the facilitator reached the specific sub-goals of each activity, e.g., whether the facilitator was able to create a process in a steering group that encouraged discussion of relevant topics, and preparation of action plans. Intervention dose was measured by counting the number of intervention activities held and the quality of participation in these activities (e.g., if action plans were followed up on). Fidelity, reach and dose were scored by the members of the research team who facilitated the intervention activities. Inter-rater reliability was qualified by comparing scorings and discussing disagreements until agreement was reached.

Fidelity, reach, and dose were weighted in terms of their estimated importance for the overall effect of the intervention. For each activity, a score on a scale from 0 to 100% was calculated. The means of these scores were used to calculate an overall degree of implementation for each work unit, ranging from 0 to 100%. For more details see [33].

## Analysis

### Analysis used to compare the intervention group with the control group

All statistical analyses were performed using the statistical software package Stata, version 16.1 (Stata Corporation, College Station, TX, USA). The analyses were conducted separately for the two sectors to better assess whether the different contexts played a role for the results. Each work unit went through three phases during the participation in the study: Control phase, intervention, and follow-up phase (see Fig. 2). Many participants responded to the questionnaire more than once (at different time points during the study).

In the mixed model, we used all the 847 responses from the 321 participants in Psychiatry and the 891 responses from the 348 participants in the PPS. The levels in the mixed model are nested in the order of work units, participants within each unit, and finally in the ten time points (contributing to the residual variance). As the data from psychiatry and the PPS were analysed separately, sector is not a part of the multi-level model.

A mixed model was used to analyse all outcomes by taking into account the between-work unit variation and the between-participant variation within a work unit. We also looked at whether there was any variation in a participant’s reports and whether there was a time factor further within participants as well as between time variation. The Akaike Information Criterion was used to select a model with a suitable variance covariance structure for the model. The fixed effects include the study phase and the study period as factors. The interaction between these was tested using the likelihood ratio test. The effect of time (trend) was also checked. The marginal effect of each study phase and a comparison of the effect with respect to the control phase were reported. If a statistically significant interaction or trend was found, we interpreted the reported marginal effect and comparisons carefully. The principal model assumptions and the normality and homogeneity of the residuals were checked by visual inspection of the diagnostic plots, such as the Q-Q and scatter plot for residuals, and the fitted values. There was no indication of any violation of model assumptions. An adjusted analysis was also performed after adjusting for the baseline information about gender, age, and job position of the participant as fixed effects. A cubic spline-smoother was used for age to allow for a non-linear effect. In the PPS the “Don’t know” response to Q1, the primary outcome, was omitted from the analyses.

### Additional analysis– imputation analysis

To assess whether results were influenced by missing data, we checked how sensitive the presented results were to sample change. It should be noted that not all the observations were missing at random or completely at random. Some of the missingness was due to reasons such as being newly employed in the work unit during the study period, changing jobs, or retiring, all of which led to ‘‘not missing at random’’.

To facilitate the imputation analysis, we redefined the design so that each participant had only one observation per study phase instead of repeated measurements within the study phase. Hence, the term time period could be omitted from the model. Three observations per person were selected, one corresponding to the last study period of each phase (Control, Intervention, and Follow-up). All three outcome values along with no-missing covariates for a participant constituted a complete case scenario at the participant level (30 for psychiatry and 29 for PPS). The missing values were imputed and analysed by combining imputation and bootstrap methods. A bootstrap sample of the same size as the number of participants available for the analysis was taken, and missing values were imputed once (single imputation) using the chained equations method with the covariates gender, age, job category, and work unit. The full data after imputation were then analysed using a model with study phase, gender, age, and job category as the fixed effects and work unit and participant ID as the random effects. This was repeated 10,000 times (10,000 different bootstrap samples), and the marginal means and contrasts were estimated each time. The imputation and the analysis were performed for different scenarios of missingness such as missing only the outcome, missing only the covariates, missing both the outcome and covariate.

## Results

### Study sample

Three of the 16 work units dropped out during the intervention (one from the PPS and two psychiatric wards) mainly due to contextual factors such as too few resources and parallel change processes.

#### Psychiatric work units

In the first survey round, the questionnaire was sent out to 249 people in eight work units in psychiatry, and 77.5% (193 persons) responded. In the last survey round, the questionnaire was sent to 181 people in the six remaining work units in psychiatry, and 24.3% (44 persons) responded. Thus, the response rate in psychiatry fell from 77.5% at the beginning of the study to 24.3% at the end.

#### Prison and Probation Service

At the first survey round, the questionnaire was sent to 181 people in eight work units in the PPS, and 66.3% (120 persons) responded. In the last survey round, the questionnaire was sent out to 197 people in the seven remaining work units in the PPS, and 40.1% (79 people) responded. Thus, the response rate for the PPS fell from 66.3% at the beginning of the study to 40.1% at the end.

### Demographics of the study population

Most employees in the PPS were male (57.5%), whereas most employees in the psychiatric wards were female (87.1%). In the PPS, most employees were in the age group 41–50 (39.2%), whereas on the psychiatric wards, the age groups 31–40, 41–50, and 51–60 were nearly identical and were all about 23%. In the PPS, most employees were uniformed prison guards (86.7%). The rest of the employees were teachers, social workers, nurses, and administrative personnel. For further details, see Table 2.

**Table 2:** Prison and Probation Service: Population characteristics, *n* = 181

	n (%)
Gender	
Women	71(39.2)
Men	104 (57.5)
Missing	6 (3.3)
Age	
≤ 21–30	2 (1.1)
≤ 31–40	48 (26.5)
≤ 41–50	71 (39.2)
≤ 51–60	43 (23.8)
> 60	7 (3.9)
Missing	10 (5.5)
Type of profession	
Uniformed personal	157 (86.7%)
Non-uniformed personal	18 (9.9%)
Missing	6 (3.3%)

On the psychiatric wards, most employees were nurses (49%) and auxiliary nurses (34.9%). The remaining participants were physicians and other professionals (e.g., physiotherapists or social workers). For further details, see Table 3.

**Table 3:** Psychiatry: Population characteristics, *n* = 249

	n (%)
Gender	
Women	217 (87.1)
Men	22 (8.8)
Missing	10 (4)
Age	
≤ 21–30	28 (11.2)
≤ 31–40	58 (23.3)
≤ 41–50	59 (23.7)
≤ 51–60	57 (22.9)
> 60	36 (14.5)
Missing	11 (4.4)
Type of profession	
Physicians	7 (2.8)
Nurses	122 (49)
Nurses auxillieres	87 (34.9)
Other professionals	22 (8.8)
Missing	11 (4.4)

### Implementation degree

Most work units were able to implement the intervention to a high degree, with an average implementation degree across all work units of 73% (see Table 4). Furthermore, after the intervention activities were adjusted to better fit the work units’ resources, the implementation degree rose from an average of 54% to an average of 84%. For a better overview, we divided the implementation degree into low, medium, and high, where 0–33% is defined as low, 34–66% as medium, and 67–100% as high. All work units that used the adjusted intervention programme reached a high intervention degree (above 66%).

Due to the low variation in implementation degree, we could not perform an analysis to assess whether implementation degree moderated the effects of the main and secondary outcomes (hypothesis 2 and 3).

**Table 4:** Degree of implementation across sectors. Implementation degree in percent

Work unit	Degree of implementation in percent	Descricption
Before adjustment of the interventionen		
Psychiatric unit 1	22	Low
Psychiatric unit 2	88	High
PPS unit 1	46	Medium
PPS unit 2	62	Medium
PPS unit 3	52	Medium
Mean degree of implementation version 1	54	
After adjustement of the interventionen		
Psychiatric unit 3	78	High
Psychiatric unit 4	79	High
Psychiatric unit 5	70	High
Psychiatric unit 6	69	High
PPS unit 4	90	High
PPS unit 5	99	High
PPS unit 6	88	High
PPS unit 7	100	High
Mean degree of implementation version 2	**84**	
Mean degree of implementation in all	**73**	

### Primary and secondary outcomes– psychiatry

The databased continuous violence prevention collaboration (primary outcomes) improved statistically significantly during the intervention (see Table 5). None of the changes in the secondary outcomes were statistically significant. However, the mean differences between the control and follow-up phases increased (improvement) by more than 0.20 for the following measurements: the violence prevention climate, line managers’ prioritization of violence prevention, the extent that guidelines for the prevention of violence and threats were carried out in practice at the work unit by the employees, and employees’ self-efficacy in violence prevention.

**Table 5:** Psychiatry: Primary and secondary outcomes

Construct and topics related violence prevention	Measure	Number of observations	Mean control phase	Mean intervention phase	Mean follow-up phase	Mean differences between follow up and control phase (confidence intervals)	P-value
**Primary outcome**							
Data based continuous violence prevention collaboration	To what extent are our violence prevention efforts continuously adjusted as a result of registrations and shared experiences?	847	6.48	6.91	7.16	0.68 (0.07;1.26)	0.03*
**Secondary outcomes**							
Cooperation between line managers and employees	To what extent does the line manager and employees cooperate on the prevention of violence and threats?	844	7.50	7.63	7.48	-0.02 (-0.61;0.57)	0.95
Attention to violence prevention	To what extent does your line manager prioritize violence prevention	879	8.07	8.27	8.30	0.23 (-0.32;0.78)	0.41
	To what extent does the working environment group prioritize violence prevention?	880	7.90	8.04	8.03	0.13 (-0.39;0.66)	0.63
Actions taken to prevent violence and threats	Has there been improvements related to the prevention of violence and threats during the last three months?	841	6.04	6.54	6.13	0.09 (-0.69;0.87)	0.82
Violence prevention practices in your work unit	To what extent are guidelines for the prevention of violence and threats carried out in practice at your workplace by your line manager?	874	7.44	7.62	7.63	0.19 (-0.39;0.77)	0.52
	To what extent are guidelines for the prevention of violence and threats carried out in practice at your workplace by the employees?	872	7.21	7.50	7.62	0.42 (-0.07;0.91)	0.10
Violence prevention climate scale	Violence Prevention Climate Scale	830	27.79	27.72	28.06	0.27 (-0.98;1.50)	0.68
Additional item about violence prevention climate	We are a unit that continuously makes an effort to prevent violence and threats.	827	5.26	5.41	5.43	0.17 (-0.11;0.46)	0.22
Self-efficacy in violence prevention	To what extent do you have confidence in your colleagues’ competences to prevent violence and threats.	832	7.68	7.69	7.85	0.17 (-0.29;0.63)	0.46
Sense of safety at work	Do you feel safe when you are at work?	834	7.89	7.76	7.80	-0.09 (-0.57;0.39)	0.72
Prevalence of violence and threats	Exposure to violence during the last three months	824	1.52	1.40	1.50	-0.02 (-0.22;0.20)	0.92
	Exposure to threats during the last three months	828	2.42	2.33	2.38	-0.04 (-0.33;0.25)	0.79

All analyses are adjusted for gender, age and profession

*p < 0.05

### Primary and secondary outcomes– PPS

The primary outcome improved, but not statistically significantly (see Table 6). Nevertheless, the intervention led to a statistically significant improvement in three secondary outcomes, namely, in the additional item on violence prevention climate, the sense of safety at work, and the employees’ self-efficacy in violence prevention. Even though the improvements in the other secondary outcomes were not statistically significant, the mean differences between the control and follow-up phases increased (improvement) by more than 0.20 for the following measures: attention to violence prevention, the extent to which the guidelines for the prevention of violence and threats were carried out in practice at the work unit by the employees, and cooperation between line managers and employees.

**Table 6:** PPS: Primary and secondary outcomes

Construct and topics related violence prevention	Measure	Number of observations	Mean control phase	Mean intervention phase	Mean follow-up phase	Mean differences between follow up and control phase (confidence intervals)	P-value
Primary outcome							
Data based continuous violence prevention collaboration	To what extent are our violence prevention efforts continuously adjusted as a result of registrations and shared experiences?	755	5.47	6.01	6.19	0.72 (-0.11;1.53)	0.09
Secondary outcomes							
Cooperation between line managers and employees	To what extent does the line manager and employees cooperate on the prevention of violence and threats?	782	5.70	6.20	6.34	0.64 (-0.19;1.45)	0.13
Attention to violence prevention	To what extent does your line manager prioritize violence prevention	849	7.49	7.49	6.93	-0.57 (-1.88;0.75)	0.34
	To what extent does the working environment group prioritize violence prevention?	779	7.80	8.16	7.69	-0.11 (-1.23;0.10)	0.84
Actions taken to prevent violence and threats	Has there been improvements related to the prevention of violence and threats during the last three months?	723	4.41	5.06	4.97	0.56 (-0.54;1.65)	0.32
Violence prevention practices in your work unit	To what extent are guidelines for the prevention of violence and threats carried out in practice at your workplace by your line manager?	781	7.63	7.45	6.81	-0.82 (-1.10;0.36)	0.76
	To what extent are guidelines for the prevention of violence and threats carried out in practice at your workplace by the employees?	832	7.12	7.48	7.82	0.70(-0.23;1.64)	0.14
Violence prevention climate scale	Violence Prevention Climate Scale	839	25.35	26.17	25.24	-0.11 (-1.69;1.45)	0.88
Additional item about violence prevention climate	We are a unit that continuously makes an effort to prevent violence and threats.	800	4.18	4.72	5.15	0.96 (0.53;1.41)	0.00*
Self-efficacy in violence prevention	To what extent do you have confidence in your colleagues’ competences to prevent violence and threats.	851	7.39	7.80	7.98	0.59 (0.01:1.16)	0.04*
Sense of safety at work	Do you feel safe when you are at work?	854	7.63	7.99	8.23	0.60 (0.56;1.15)	0.03*
Prevalence of violence and threats	Exposure to violence during the last three months	843	1.44	1.29	1.28	-0.14 (-0.34;0.6)	0.16
	Exposure to threats during the last three months	845	1.92	1.84	1.88	-0.04 (-0.30;0.23)	0.79

All analyses are adjusted for gender, age and profession

*p < 0.05

The intervention also had three non-significant negative effects, the mean differences between the follow-up and control phases decreased by more than 0.20 for the following measures: the line managers’ prioritization of violence prevention, the extent to which the guidelines for the prevention of violence and threats were carried out in practice by the line manager, and employees’ self-efficacy in violence prevention.

### Exploratory analyses

#### Results of the imputations

The exploratory imputation analyses did not lead to statistically significant changes that were different from the results without imputation. The directions of the estimates also did not change, which means that the decreasing response rate probably did not introduce response bias. The results from the imputed data analysis are therefore not presented in the results section.

## Discussion

In the present study, a comprehensive and participatory intervention aimed at improving violence prevention was tested in two high-risk occupations. With regard to hypothesis 1, the intervention led to a statistically significant improvement in the primary outcome on the psychiatric work units but not in the PPS work units. However, the intervention did lead to statistically significant improvements in several secondary outcome measures in the PPS work units. With regard to hypotheses 2 and 3, the overall high implementation degree indicates that most work units were able to carry out the intervention as planned. Due to low variation in the degree of implementation, we could not test whether the degree of implementation had any the effect on the primary and secondary outcomes. Finally, with regard to hypothesis 4, we did not find an indication of a ceiling effect, because it was the sector with the higher baseline measure (psychiatric wards) where we found a significant improvement in the primary outcome.

### Interpretation

We unexpectedly found that the intervention only led to a statistically significant improvement in the primary outcome on the psychiatric work units and not in the PPS work units.

An explanation may be that registrations of incidences of violence and threats in the PPS were analysed at a different organisational level than on the psychiatric wards. In the PPS, the registrations were less accessible to employees. This may have led to uncertainty among PPS employees when answering the question that asked about continuous work on violence prevention based on the registrations. This weakened the statistical power of the analyses of the PPS data and may explain why the mean differences between the follow-up and control phases were not statistically significant despite improvements in these two phases. In both sectors, we found improvements in several secondary outcomes measures that were not statistically significant. However, conclusions should not be based solely on statistical significance. The size and direction of the estimates are also important [39], especially when studies are not powered for the specific analyses, which was the case in the present study.

We conducted a power analysis prior to the study that estimated a need for 300 employees to achieve statistically significant results. We assumed a drop-out rate of 25% during the questionnaire rounds, meaning that we would need to recruit approximately 400 employees, but this goal was not reached. Due to the drop-out of three work units and a decrease in the response rate during the ten survey rounds, the sample was smaller than expected, further reducing the statistical power of our study.

Nevertheless, the results from both sectors indicate that the intervention led to several improvements in the different measures of violence prevention. This is supported by the results of qualitative process data presented in Karlsen et al., 2022 [40] and the insights gained from the qualitative interviews with employees describing their experiences of preventing violence [19] that were collected as part of this research project. The process evaluation of the study (Karlsen et al., 2022) shows that the intervention led to considerable preventive activities: employees and line managers developed 293 suggestions and 92 action plans to prevent workplace violence. The most frequently used action plan topics focused on aspects like de-escalating techniques, improved communication between employees in different shift groups, and more focus on the instruction of new colleagues, including temporary workers. An example of an action plan implemented by one of the work units in PPS involved standardizing the signage on doors. This initiative aimed to enable replacements or substitutes from other departments to easily recognize inmates’ affiliations. By doing so, the goal was to prevent inadvertent door openings to inmates associated with rival gangs, reducing the likelihood of conflicts or assaults. Another example of an action plan is from a psychiatric work unit that initiated an action plan on improving the steering of their daily morning meetings and time-out-sessions in the afternoon to include the most relevant information for violence prevention. The vast amount of the developed action plans and their tailored approach as illustrated in the two examples shows that although the quantitative results reported here are limited and mixed, the intervention led to actual changes in violence prevention activities.

With regard to hypotheses 2 and 3 concerning the associations between implementation and primary and secondary outcomes, we found that the variation in implementation degree was insufficient to allow analysis. The high degree of implementation for the majority of the 13 work units illustrates that most work units were able to carry out the intervention as planned although psychiatric wards and PPS work units are known to have high work demands [41].

It is an interesting result that the intervention improved the employees’ sense of feeling safe at work. There are several ways in which the intervention might have been effective to increase the sense of safety at work. Firstly, and probably most important, during the steering group meetings, many safety issues were addressed through action plans [40]. This may have resulted in employees perceiving that the work unit was consistently making efforts to prevent violence and threats (significant outcome of the intervention). Secondly, the perceived effort may have created a greater confidence in colleagues’ abilities to prevent violence and threats (significant outcome of the intervention). These mechanisms may have led to employees feeling more secure when going to work.

It has been argued that complex interventions, specifically participative interventions (such as the IVP) can work through different mechanisms [42, 43]. This might also have happened here. The intervention consisted of a process with discussions and the development of a variety of action plans. While we assumed that “feeling safe” could only be achieved by tangible changes in the work environment that made the workplace actually safer, it might be that discussions about existing safety and new measures and reassurance about colleagues supporting each other in keeping each other safe lead to the experience of feeling safer.

The employees were involved in every aspect of the intervention, from problem analysis, to generating suggestions for improvement, to trying out solutions in practice, to making adjustments if needed. This participatory process may have increased the employees’ ownership of the intervention, thus helping to ensure commitment and acceptance of the intervention activities and suggestions generated by the intervention [44–46]. Nevertheless, contextual factors such as resource availability, parallel change processes, culture of participation, and change in management were found to hinder the implementation of the intervention in a few units [47]. Similar results are reported in a study of the Safewards model in public mental health facilities that found that implementation was dependent on management support and willingness of nursing staff to engage with the trial and undertake new and additional activities [28]. This is in line with more general observations from organisational level occupational health interventions that find that organisational context, available resources, recruitment, employees’ attitudes towards the intervention, and management support often affects implementation and outcomes [48, 49].

The psychiatric work units were able to improve the primary outcome despite starting with higher baseline measures, thereby disproving our fourth hypothesis about a ceiling effect. However, the ceiling effect might have played a role, as we did not find any other significant changes in the psychiatric work units, while we found three significant changes in the PPS work units, which started from a lower baseline level in almost all measures. Therefore, our hypothesis that it is more difficult to achieve improvements when starting from a high baseline level might still be relevant.

### Strengths and limitations

Our study has some important strengths. While the intervention approach was standardised, each work unit developed and implemented their specific action plans. In that way, we supported work units with a structure that allowed them to find their own solutions instead of suggesting that they followed a standardised procedure that did not take local needs and possibilities into account [50]. In addition, the intervention approach encouraged and supported work units to systematically implement several action plans and not just one specific activity [40]. Thereby, this intervention approach was able to address the complexity of violence prevention. Another strength is that we assessed the degree of implementation and could show that most work units were able to implement the intervention and that the detected effects were therefore most likely caused by the intervention.

We used a stepped wedge cluster-randomised study design that enabled us to let all work units receive the intervention, which made the recruitment process easier [51]. Another advantage of this design is that each cluster switches from control phase to intervention phase at different time points, making it possible to examine time effects [52]. The statistical power of the study was increased by each work unit being assessed during both the control phase and the intervention phase, thus keeping the number of work units smaller than would be necessary in a traditional randomised controlled trial [53].

However, the study also has some limitations. Despite the advantages, the stepped wedge study design also has disadvantages. We asked participants to answer the same questionnaire ten times during a period of 27 months. Although missing data are a common problem in organisational research [54], this design might have contributed to a decline in response rates. In addition, the rather long study period inevitably led to a certain amount of missing data due to maternity leave, sickness absence, employees leaving the work unit, or retirement. However, the imputation analysis showed that the direction of the estimates did not change when missing data were imputed. It would have been desirable to use more items to measure the different outcomes. The reason why we applied single items (except for the violence prevention climate scale) was to investigate many aspects of violence prevention while still ensuring a high response rate. Single items have the advantage of making answering the questions easier for the respondent and shortening the time to fill out the questionnaires [55], which may have ensured that the response rate did not decrease further.

The recruitment process may represent a limitation. Work units had to sign up for participation and were not selected at random or representatively. Thus, selection bias cannot be ruled out, which challenges the external validity of the results. However, the stepped wedge design made it possible to use the same group of interested and potentially selected work units under control, implementation, and follow-up conditions, thereby eliminating the risk of comparing work units with high interest in the intervention with work units with low interest in the intervention. In addition, there were no indications that the participating work units were different from comparable work units in the two sectors in relation to risk of violence and threats or cooperation between staff and management.

An additional limitation is that outcome data were entirely based on self-reported measures, which may have caused common method variance, potentially inflating the associations between the examined variables [56]. Yet, as mentioned before, our additional process evaluation showed that work units actively developed and implemented action plans, thereby making it more likely that the effects were caused by actual improvements in violence prevention.

Even though we carried out a detailed assessment of the implementation of the intervention, it was a weakness that the facilitators themselves evaluated the delivery of the intervention activities. However, we have tried to make the implementation assessment as objective as possible (e.g., assessing whether the activity was delivered or not).

### Implications and generalisability

The complexity and interplay of the numerous risk factors for violence at work from clients and patients requires comprehensive violence prevention interventions, not isolated activities. The practical implication of this study is that a participatory and comprehensive intervention could increase cooperation in preventing work-related violence and threats in high-risk workplaces. Comprehensive violence prevention may not only contribute to better quality collaboration, but also protect employees’ health and improve organisational outcomes such as lower turnover and less sickness absence.

## Conclusion

Most work units were able to carry out the intervention as planned. The intervention showed mixed results for all outcomes, indicating that the intervention could be working through different mechanisms due to its participative nature that allow for considerable variation in the preventive actions implemented.

While results are promising, they should be interpreted cautiously until more robust evidence is available. Nevertheless, this participatory and comprehensive approach could be a viable way of improving the prevention of work-related violence and threats in high-risk workplaces.

## Data Availability

To protect the privacy of students and ensure the confidentiality of the data, the datasets used and analysed during the study are not publicly available but can be gained from the corresponding author on reasonable request.
