# Correction: A participatory and comprehensive intervention to improve violence prevention in two high-risk occupations: effect and process evaluation of a stepped wedge cluster randomised trial

**DOI:** 10.1186/s12889-024-18632-5

**Published:** 2024-05-06

**Authors:** Lars Peter Andersen, S. Jaspers, D. Andersen, I. Karlsen, B. Aust

**Affiliations:** 1 Department of Occupational Medicine, Danish Ramazzini Centre, University Research Clinic, Goedstrup Hospital, Hospitalsparken 15, 7400 Herning, Denmark; 2 The National Research Centre for the Working Environment, Lersø Parkallé 105, 2100 Copenhagen Ø, Denmark

**Correction:** **BMC Public Health 24, 1043 (2024)**

**https://doi.org/10.1186/s12889-024-18527-5**

The original publication of this article [1] contained an incorrect version of Table 5, which was a duplicate of Table 6. The incorrect and correct version of Table 5 are published in this correction article. The original article has been updated.

**Incorrect table 5**

**Table:** 

**Construct and topics related violence prevention**	**Measure**	**Number of observations**	**Mean control phase**	**Mean intervention phase**	**Mean follow-up phase**	**Mean differences between follow up and control phase (confidence intervals)**	***P*** **-value**
**Primary outcome**							
Data based continuous violence prevention collaboration	To what extent are our violence prevention efforts continuously adjusted as a result of registrations and shared experiences?	755	5.47	6.01	6.19	0.72 (-0.11;1.53)	0.09
**Secondary outcomes**							
Cooperation between line managers and employees	To what extent does the line manager and employees cooperate on the prevention of violence and threats?	782	5.70	6.20	6.34	0.64 (-0.19;1.45)	0.13
Attention to violence prevention	To what extent does your line manager prioritize violence prevention	849	7.49	7.49	6.93	-0.57 (-1.88;0.75)	0.34
	To what extent does the working environment group prioritize violence prevention?	779	7.80	8.16	7.69	-0.11 (-1.23;0.10)	0.84
Actions taken to prevent violence and threats	Has there been improvements related to the prevention of violence and threats during the last three months?	723	4.41	5.06	4.97	0.56 (-0.54;1.65)	0.32
Violence prevention practices in your work unit	To what extent are guidelines for the prevention of violence and threats carried out in practice at your workplace by your line manager?	781	7.63	7.45	6.81	-0.82 (-1.10;0.36)	0.76
	To what extent are guidelines for the prevention of violence and threats carried out in practice at your workplace by the employees?	832	7.12	7.48	7.82	0.70(-0.23;1.64)	0.14
Violence prevention climate scale	Violence Prevention Climate Scale	839	25.35	26.17	25.24	-0.11 (-1.69;1.45)	0.88
Additional item about violence prevention climate	We are a unit that continuously makes an effort to prevent violence and threats.	800	4.18	4.72	5.15	0.96 (0.53;1.41)	0.00*
Self-efficacy in violence prevention	To what extent do you have confidence in your colleagues’ competences to prevent violence and threats.	851	7.39	7.80	7.98	0.59 (0.01:1.16)	0.04*
Sense of safety at work	Do you feel safe when you are at work?	854	7.63	7.99	8.23	0.60 (0.56;1.15)	0.03*
Prevalence of violence and threats	Exposure to violence during the last three months	843	1.44	1.29	1.28	-0.14 (-0.34;0.6)	0.16
	Exposure to threats during the last three months	845	1.92	1.84	1.88	-0.04 (-0.30;0.23)	0.79

All analyses are adjusted for gender, age and profession

*p<0.05

**Correct table 5**

**Table:** 

**Construct and topics related violence prevention**	**Measure**	**Number of observations**	**Mean control phase**	**Mean intervention phase**	**Mean follow-up phase**	**Mean differences between follow up and control phase (confidence intervals)**	***P*** **-value**
**Primary outcome**							
Data based continuous violence prevention collaboration	To what extent are our violence prevention efforts continuously adjusted as a result of registrations and shared experiences?	847	6.48	6.91	7.16	0.68 (0.07;1.26)	0.03*
**Secondary outcomes**							
Cooperation between line managers and employees	To what extent does the line manager and employees cooperate on the prevention of violence and threats?	844	7.50	7.63	7.48	-0.02 (-0.61;0.57)	0.95
Attention to violence prevention	To what extent does your line manager prioritize violence prevention	879	8.07	8.27	8.30	0.23 (-0.32;0.78)	0.41
	To what extent does the working environment group prioritize violence prevention?	880	7.90	8.04	8.03	0.13 (-0.39;0.66)	0.63
Actions taken to prevent violence and threats	Has there been improvements related to the prevention of violence and threats during the last three months?	841	6.04	6.54	6.13	0.09 (-0.69;0.87)	0.82
Violence prevention practices in your work unit	To what extent are guidelines for the prevention of violence and threats carried out in practice at your workplace by your line manager?	874	7.44	7.62	7.63	0.19 (-0.39;0.77)	0.52
	To what extent are guidelines for the prevention of violence and threats carried out in practice at your workplace by the employees?	872	7.21	7.50	7.62	0.42 (-0.07)	0.10
Violence prevention climate scale	Violence Prevention Climate Scale	830	27.79	27.72	28.06	0.27 (-0.98;1.50)	0.68
Additional item about violence prevention climate	We are a unit that continuously makes an effort to prevent violence and threats.	827	5.26	5.41	5.43	0.17(-0.11;0.46)	0.22
Self-efficacy in violence prevention	To what extent do you have confidence in your colleagues’ competences to prevent violence and threats.	832	7.68	7.69	7.85	0.17 (-0.29;0.63)	0.46
Sense of safety at work	Do you feel safe when you are at work?	834	7.89	7.76	7.80	-0.09(-0.57;0.39)	0.72
Prevalence of violence and threats	Exposure to violence during the last three months	824	1.52	1.40	1.50	-0.02 (-0.22;0.20)	0.92
	Exposure to threats during the last three months	828	2.42	2.33	2.38	-0.04 (-0.33;0.25)	0.79

All analyses are adjusted for gender, age and profession

*p<0.05
